# Systemic hormone therapy and dementia: A nested case-control and co-twin control study

**DOI:** 10.1016/j.maturitas.2022.04.007

**Published:** 2022-05-25

**Authors:** Laura Ekstrøm Løkkegaard, Mikael Thinggaard, Marianne Nygaard, Jesper Hallas, Merete Osler, Kaare Christensen

**Affiliations:** a Epidemiology, Biostatistics and Biodemography, Department of Public Health, University of Southern Denmark, J. B. Winsløws Vej 9B, 5000 Odense C, Denmark; b The Danish Twin Registry, Department of Public Health, University of Southern Denmark, J. B. Winsløws Vej 9B, 5000 Odense C, Denmark; c Danish Aging Research Center, Unit of Epidemiology, Biostatistics and Biodemography, Department of Public Health, University of Southern Denmark, J. B. Winsløws Vej 9B, 5000 Odense C, Denmark; d Clinical Pharmacology, Pharmacy and Environmental Medicine, Department of Public Health, University of Southern Denmark, Odense University Hospital, J. B. Winsløws Vej 19, 5000 Odense C, Denmark; e Center for Clinical Research and Prevention, Bispebjerg and Frederiksberg Hospitals, Nordre Fasanvej 57, 2000 Frederiksberg, Copenhagen, Denmark

**Keywords:** Postmenopausal hormone therapy, Twins, Register data, Population-based study

## Abstract

**Objective:**

The effect of systemic hormone therapy (HT) on dementia risk is unclear. Our aim was to investigate the association between HT and dementia.

**Study design:**

This register-based study consists of a nested case-control study and a co-twin control design, which controls for familial confounding, including shared genetics.

**Main outcome measures:**

Through Danish national registries from 1995 to 2011, we identified: a) 2700 female singletons with incident dementia and 13,492 matched controls; b) 288 female twins with incident dementia and co-twins without dementia. Data on HT and education were retrieved, and analyses were performed using conditional logistic regression and McNemar’s χ^2^-test. HT use decreased dramatically after the Women’s Health Initiative study results were published in 2002, and the analyses were stratified accordingly to account for potentially different HT user characteristics.

**Results:**

The odds ratio (OR) for the association between systemic HT and dementia was 1.05, 95% CI = [0.93–1.19] in singletons and 2.10, 95% CI = [0.99–4.46] in twins. A statistically significant association was found for systemic HT before 2003 in both populations, with an OR of 1.14, 95% CI = [1.01–1.28] in singletons and an OR of 2.20, 95% CI = [1.04–4.65] in twins.

**Conclusion:**

Using Danish nationwide registries and controlling for education and for familial factors in a sub-sample, systemic HT was found to be associated with increased dementia risk if used before 2003, when HT was more commonly prescribed.

## 1. Introduction

Dementia is a worldwide challenge. An estimated forty-seven million people were living with dementia by 2015, and this number is expected to increase dramatically within the next three decades [1]. The incidence of dementia is growing globally, but a decline in age-specific dementia incidence has been suggested in several studies from, among others, the United States and Holland [2,3]. A Danish population-based register study has also added evidence to these findings by showing a decrease in age-specific dementia incidence rates, but an increase in prevalence [4].

The underlying mechanisms of the disease are likely multiple and accumulate over the life course with both environmental (e.g., smoking and social isolation) and genetic (e.g., the *APOE* e4 allele) factors at play [1,5]. However, there is a well-documented sex difference in dementia prevalence [6,7].

Several observational studies have reported a protective effect of hormone therapy (HT) on dementia, but clinical studies and other observational studies have not been able to corroborate these findings [8–11]. In 2002, the Women’s Health Initiative (WHI) published a seminal randomized clinical trial, which found that systemic HT led to risks outweighing the benefits of disease prevention. This finding changed the view on HT worldwide and led to a marked decrease in HT use among postmenopausal women [12]. Level of education has also been hypothesized to influence HT use as higher education was found to be associated with long-term use and higher compliance [13]. Differences in education, and socioeconomic status more generally, between HT users and non-users have been hypothesized to be a factor in the contradictory findings of clinical trials and observational studies [14,15].

Our aim was to investigate the association between HT and dementia using the Danish health registries. We performed two studies: A nested case-control study and a co-twin control study. Both studies were adjusted for education. The co-twin control study design enabled us to control for unobserved familial confounding as twins have a shared childhood environment and are matched either fully or partly on genetic factors depending on zygosity [16].

## 2. Methods

The nested case-control study and the co-twin control study were based on the following Danish nationwide registries: The Danish Civil Registration System, The Danish Twin Registry, the Danish National Prescription Registry, the Danish National Patient Register, and Statistics Denmark. A description of these registries is provided in Supplementary Table S1.

### 2.1. Singleton population

**Fig. 1.**
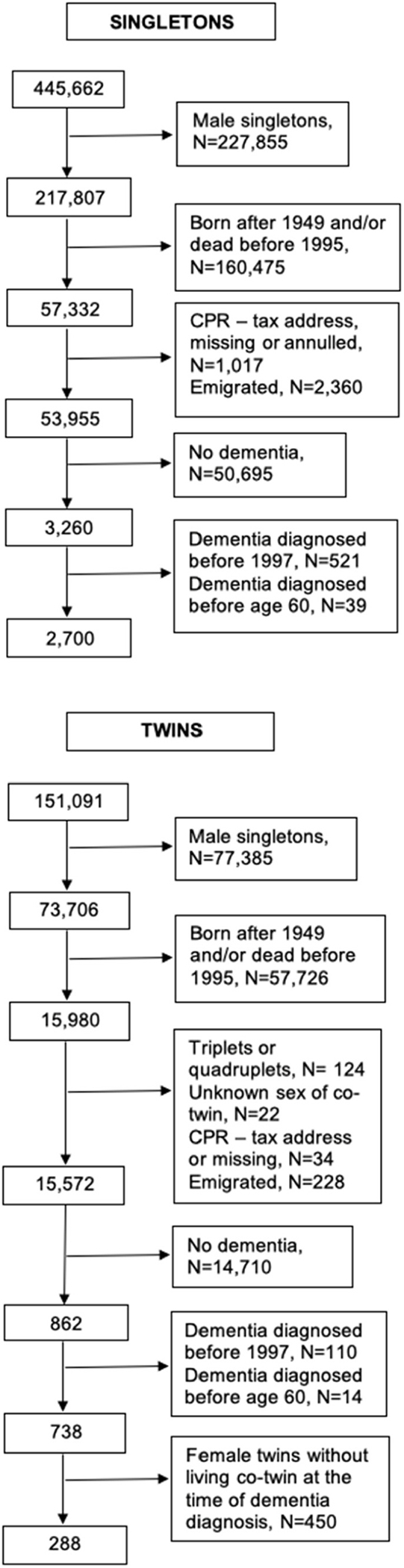
Selection process and exclusion criteria for the singleton cases and twin cases, respectively. Abbreviation: CPR, personal identification number from the Danish Civil Registration System.

A 5% random sample of female singletons from the general Danish population was identified through the Danish Civil Registration System. The singleton study population was restricted to individuals without a twin, born before 1950, and alive by 1995. Thus, the study population consisted of singleton women aged 45+ in 1995 when drug exposure information became available. Further criteria required that cases had to have received the first dementia diagnosis after 1997 and that they were at least 60 years of age at the time of the diagnosis, resulting in a total of 2700 cases (Fig. 1). The two-year lag period for dementia diagnosis was included to minimize inclusion of prevalent dementia patients, while the age limit for dementia diagnosis of 60 years was an attempt to exclude heritable forms of early onset dementia. Each case was matched with five female singletons born the same year as the case and living without dementia at the time of the case’s dementia diagnosis. All except two cases could be matched with five controls, resulting in a total of 13,492 controls.

### 2.2. Twin population

The twin study population was identified through the Danish Twin Registry and was constricted to the criteria shown in Fig. 1. As a result, the twin study population consisted of 288 twin cases with a living co-twin without a dementia diagnosis on the date of the dementia diagnosis as their control.

### 2.3. Dementia outcome

Information on dementia diagnoses was obtained from the Danish National Patient Register. A diagnosis with one of the following ICD-codes was considered a dementia case: ICD-8: 290.00–290.99 and ICD-10: F00.0–F03.9; G30.0–G30.9. The index date was defined as the date of the first dementia diagnosis. Date for end of case inclusion was 31/12–2011.

The validity of dementia diagnoses from ICD-10 codes from the Danish National Patient Register has shown to be high (85.8% correctly diagnosed), although Alzheimer’s Disease (AD) is under-registered and division into dementia subtypes has a low validity [17].

### 2.4. Hormone therapy exposure

Exposure was based on prescriptions from the Danish National Prescription Registry and included estrogen and progestogen hormone therapy sold in Denmark from 1995 until index date or end of study period in 2011, whichever came first. Prescriptions were disregarded if redeemed less than a year before the index date to include a latency period or after the index date as outcome came before the exposure. The following HT regimens were included: Continuous estrogen, cyclic progestogen, continuous combined estrogen and progestogen, and cyclic combined estrogen and progestogen.

Systemic HT was defined as either oral or transdermal, and systemic HT users were considered as such if one or more prescriptions for systemic HT were redeemed during the study period (1995–2011). Vaginal estrogen users and users with an intrauterine device only were excluded, as they may be an intermediate group having limited systemic effect of HT. The following ATC-codes were included in this study:

G03C A03, G03C A04, G03C A53, G03C A57, G03C B01, G03D A02, G03D A04, G03D C02, G03D C03, G03F A01, G03F A12, G03F A15, G03FA17, G03F B01, G03F B05, G03F B06, G03F B09, G03F B11, and G03H B01.

### 2.5. Education

Education was defined according to standard number of schooling years in 1980 [18]. Study participants were divided into two groups according to length of education (≤ 7 years or >7 years).

### 2.6. Statistical analysis

The association between HT and dementia was performed as a nested case-control study in which cases and controls were drawn from the above-mentioned singleton population. Excluding vaginal estrogen users and users with an intrauterine device resulted in 2235 cases (female singletons with dementia) who were matched with five female singletons born the same year and alive and with no dementia diagnosis in the Danish National Patient Registry by the time of the case’s index date. This led to 11,028 controls. The analysis in the nested case-control study was performed using conditional logistic regression. Time-dependent Cox regressions were also performed on the singleton population to ensure the randomness of the control selection did not impact the results, as results from these analyses were like those derived from conditional logistic regression.

The twin study population consisted of Danish female twin pairs of which one twin had received a dementia diagnosis and the co-twin was alive and without dementia on the index date. Vaginal estrogen users and users with an intrauterine device were excluded, resulting in 204 twin pairs. Another intrapair analysis of complete twin pairs discordant on both systemic HT and dementia status was also performed and further split according to zygosity to evaluate whether the results differed between monozygotic and dizygotic twin pairs. The association between systemic HT and dementia in the co-twin control design was estimated using McNemar’s χ^2^-test and conditional logistic regression.

#### 2.6.1. Age at diagnosis and education adjustments

An analysis was performed using conditional logistic regression to examine the influence of participants with missing or unknown educational status as a result of their not being included in Statistics Denmark’s educational dataset due to their advanced age. In this analysis, cases were split into two groups according to age at first dementia diagnosis (below or above age 80) and an adjustment for education was made by only including participants with information on education.

#### 2.6.2. Sensitivity analyses

Two sensitivity analyses were performed in both studies:

- Firstly, a sensitivity test was done by categorizing 1–2 HT prescription users as non-users. The intention was to test the influence of 1–2 HT prescription users on the risk estimate, as it can be argued that they accumulate too small a dosage to be clinically relevant, thus risking misclassification by including them in the user group. Those registered with 1–2 HT prescriptions in 1995 were not categorized as non-users as they may have had an HT use before the registry was established.
- Secondly, a sensitivity test was performed in which all dementia cases were included regardless of age at diagnosis to test the soundness of excluding possible heritable forms of early onset dementia.

#### 2.6.3. Hormone therapy use before or after the WHI study

An analysis was performed to examine the influence of alterations made in national HT guidelines following the publication of the WHI study in 2002. For analysis purposes these alterations were considered effective as of 1/1–2003. HT users were divided according to time of exposure: HT use before 1/1–2003 or HT use continued or initiated after 1/1–2003.

When stratifying according to HT use before the WHI study publication, 2305 singleton cases and 218 twin cases were included in the analysis (1995–2003). This number differs from the number of individuals in the primary analyses (1995–2011) as some singletons (*N* = 70) and twin pairs (*N* = 14), that were non-users before 2003, were excluded after 2003 due to use of vaginal estrogen or intrauterine device only.

Systemic HT users before 2003 had a follow-up time from 1997 to 2011, whereas users after 2003 had a follow-up time from 2005 to 2011. The same criteria as mentioned in 2.4 applied with systemic HT having to be initiated more than a year before the dementia diagnosis.

User status was defined as never user, previous user (before 2003), or current user (2003–2011) to illustrate from which user group the risk estimates derived.

All analyses were performed using STATA 15.1.

## 3. Results

### 3.1. Systemic HT and dementia risk in singletons

The descriptive characteristics were distributed evenly between the 2235 cases and 11,028 controls in the singleton study population (Table 1). Nearly half of the study participants among both cases and controls were too old to be included in the education registry. When examining the association between systemic HT and dementia in the singleton study population, we found an Odds Ratio (OR) of 1.05 with a 95% Confidence Interval (CI) of [0.93–1.19] (Table 2). No indication of a cumulative dose-response association was found, as the OR did not increase with increasing cumulative dosage. The tendency of a slightly increased risk of dementia for systemic HT in the singleton study population persisted when stratifying according to age at dementia diagnosis (below or above age 80) and adjusting for education (Table 2). The alterations in exposure or outcome definitions in the two sensitivity analyses did not change the OR appreciably (Table 2). A statistically significant increase in dementia risk was observed (OR = 1.14, 95% CI = [1.01–1.28]) for systemic HT use before 2003, but no cumulative dose-response association was found (Table 3). The statistically significant risk estimate seems to derive from the previous user group, who had a higher OR of 1.20, 95% CI = [0.98–1.47] compared to the current users (OR = 1.00, 95% CI = [0.82–1.21]). Cox regression showed similar results to those from the nested case-control study, ensuring that selection of the controls did not have an impact on the results.

### 3.2. Systemic HT and dementia risk in twins

The twin study population consisted of 204 female twin pairs discordant for dementia at the index date. Systemic HT use was registered in 20.1% of the cases compared to 14.7% of the controls (Table 1). Among both cases and controls, nearly one third had missing or unknown information on education.

The association between systemic HT and dementia in the twin population showed a borderline statistically significant increase in risk of dementia with OR = 2.10, 95% CI = [0.99–4.46], (Table 2). The tendency of increased risk, as seen in the singleton study population, attenuated in the twin study population when stratifying by age of dementia and adjusting for education (Table 2).

A statistically significant increase in dementia risk was observed (OR = 2.20, 95% CI = [1.04–4.65]) for systemic HT use prior to 2003 (Table 3). The same tendency, as in singletons, was seen with the risk estimate deriving from previous users. The McNemar test confirmed the results from the conditional logistic regression.

Of the 31 case-control twin pairs with discordant exposure status, there were 21 pairs where the one having used systemic HT was also the one having received a dementia diagnosis, whereas in only 10 pairs the twin without dementia was the twin exposed to systemic HT (Table 4). This distribution resulted in a doubling of dementia risk if systemic HT user (OR = 2.10, 95% CI = [0.95–4.99]). Stratifying the analysis according to zygosity disclosed a larger effect in monozygotic twins than in dizygotic twins; however, there was no significant difference between the ORs in these small samples.

## 4. Discussion

### 4.1. Main findings

In this observational study, we found an overall borderline statistically significant tendency towards an association between systemic HT use and dementia in both singleton and twin analyses. This tendency persisted in the singleton study population when stratifying according to age at dementia diagnosis and adjusting for education, but no cumulative dose-response association was detected. However, we did find a statistically significant increased risk of dementia for systemic HT use in both singletons and twins when HT was used before 2003 – a time where HT was more commonly prescribed to postmenopausal women.

The WHI study published in 2002 found that systemic HT led to risks outweighing benefits for disease prevention [12]. In Denmark, national guidelines on HT treatment for menopausal symptoms were subsequently altered, and HT was now suggested to be kept at a minimum dosage for a minimum period of time. The WHI publication and the following alteration in guidelines may have caused the 65% reduction in systemic HT DDD observed in Denmark after 2002 [19]. This could indicate that our findings are driven by an exclusion of the most fragile women after 2002, as the WHI study showed adverse health effects of HT. Another probable explanation for the discrepancy in results before and after 2002 could be that the follow-up time after the guideline alteration is too short for a possible increase in dementia risk to be detected.

### 4.2. Comparison with other studies

HT use and dementia risk is a research field that has presented contradictory findings for decades. Many observational studies have shown a reduced risk of dementia among HT users [8]. The placebo-controlled trial Women’s Health Initiative Memory Study (WHIMS) contradicted these findings as it showed an increase in risk of probable dementia in HT users [20,21]. These findings were supported by a recent, large, nationwide case-control study from Finland also reporting an increased risk of AD in women using systemic HT [9]. Our study cautiously aligns with these findings, as we observed an increased risk of dementia, especially for HT users before 2003.

A follow-up report from the WHI 18 years later showed reduced risk of dementia related death for HT users [22]. In addition, a Finnish observational study found a lower risk of death from AD and vascular dementia with the biggest reduction in risk in the latter dementia type [23]. These findings stand in contrast to the otherwise reported increased risk of dementia from WHIMS and Finland but should be interpreted with caution due to underreporting of dementia related deaths, competing risks and residual confounding. For instance, differences in socioeconomic status have previously been considered a possible confounder of the positive effect of HT on dementia [24]. In this study, we used education as a proxy for socioeconomic status and found that it did not alter the results when adjusting for education. This finding differs from findings from American and some Nordic studies, but aligns with a Danish, register-based cross-sectional study, as Danish observational studies on HT may not have suffered from strong confounding from socioeconomic status due to a differently structured public health system [25].

### 4.3. Strengths and limitations

Our register-based study lacks information on lifestyle, e.g., smoking, alcohol, and body mass index. This information could have highlighted any baseline differences in HT users compared to non-users, especially when stratifying according to HT use before or after 2003. Baseline information could also have illustrated the distribution of known risk factors for dementia, e.g., smoking, hypertension, and low education [26,27]. We detected no difference in the distribution of educational status among cases and controls, but we did have a high number of individuals with missing or unknown educational status due to the oldest study participants not being in the education registry. This limits the clarification of the relation between education and dementia, as educational level has previously been found to have an influence on dementia risk in a previous Danish study [28]. Additionally, we have no available information on menopausal age and age at initiation of HT for the oldest cohorts in the Danish registries. This information would have been useful in investigating whether HT was indeed prescribed as treatment for menopausal symptoms and to illuminate the timing hypothesis of HT initiation [29]. However, we examined age when first systemic HT was prescribed in the youngest birth cohorts (1940–1944). In these, 47.7% had at some point in the observation time redeemed at least one prescription for systemic HT. Of these, 98.3% redeemed their first systemic HT prescription before age 60 and 82.9% before age 55, which indicates that for far the majority of users, the initiation was around the time of menopause, as the average age at menopause onset in European countries is 50–53 years [30].

It is hypothesized that systemic estrogen alone is responsible for an increased risk of AD, but that progestogens could possibly potentiate this increased risk [9]. We were not able to stratify according to HT regimen and, consequently, we have listed the risk estimates for systemic HT only. The majority of HT users in our study used an estrogen alone or in combination with a progestogen. Less than 1% had cyclic progestogen prescriptions only, which may not have prescribed for menopausal symptoms. As there were very few users of this HT regimen, this should not alter the risk estimates. Also, we were only able to address cumulative dosage within the observation time of this study as estimation of the total HT use over a lifetime was not possible, due to HT prescriptions not being available before 1995.

As in WHIMS, stratification according to dementia subtype was hindered due to lack of statistical power, and we were unable to include recipients of cholinesterase inhibitors to increase the number of cases. However, dementia diagnoses registered in the Danish National Patient Registry are of high validity and are considered suitable for epidemiological research, whereas division according to subtype is considered less reliable in Danish registries [17].

Even though a one-year latency period between HT and dementia diagnosis was included, the risk of reverse causation still exists in principle as some women could initiate HT to reduce beginning cognitive decline, thus making early dementia symptoms the reason for HT use and not the result thereof. Additionally, as this is an observational study, other unknown sources of residual confounding cannot be ruled out.

Among the strengths of our study are the linkages of nationwide registries using the unique personal identification number in combination with data from the universal health care. This provided an excellent setting for examining the association between HT and dementia, while allowing us to control for education. The nested case-control study was based on a large, random sample from the Danish population, which, in addition to the minimization of selection bias, is considered a strength due to the large sample size.

A major strength is the co-twin control design, as it enables us to control for shared genetics and early childhood environment by considering that monozygotic twins are an almost perfect genetic match, while dizygotic twins share on average half of their segregating genes, like other full siblings, and that almost all twins share common up-bringing, i.e., a shared family environment. The association between systemic HT and dementia showed a tendency to be stronger in the twin study population than in the singleton study population. Dementia is a disease with a high heritability [31], so when controlling for shared genetic factors, we expected the signal to be more accurate [32].

### 4.4. Implications of findings

In this register-based study, we found evidence of an increased risk of dementia for systemic HT use before 2003 in both singletons and twins, but no evidence of a cumulative dose-response association. Although causality cannot be ruled out, these findings may be a result of confounding by indication, as alterations in national HT guidelines from 2003 may have led to exclusion of the most fragile women.

## Supplementary Material

Supplementary Material

## Figures and Tables

**Table 1:** Descriptive characteristics of the singleton and twin populations.

	Singletons		Twins	
	Cases (*N* = 2235)	Controls (*N* = 11,028)	Cases (*N* = 204)	Controls (N = 204)

Zygosity				
Monozygotic			68 (33.3)	68 (33.3)
Dizygotic			123 (60.3)	123 (60.3)
Unknown			13 (6.4)	13 (6.4)
HT use, N (%)				
Non-user	1825 (81.7)	9193 (82.3)	163 (79.9)	174 (85.3)
Systemic	410 (18.3)	1974 (17.7)	41 (20.1)	30 (14.7)
Number of HT prescription, median (IQR)	19 (5–35)	17 (5–34)	15 (3–29)	33 (8–41)
DDD for HT users, median (IQR)	1344 (300–2772)	1300 (252–2688)		
Age at first dementia diagnosis, median (IQR)	83 (78–87)		79 (74–84)	
Age at first dementia diagnosis, N (%)				
60–69 years	152 (6.8)		107 (52.5)	
70–79 years	592 (26.5)			
80–89 years	1128 (50.5)		97 (47.5)	
90+ years	363 (16.2)			
Education, N (%)				
≤ 7 years	667 (29.8)	3296 (29.5)	72 (35.3)	80 (39.2)
> 7 years	540 (24.2)	2679 (24.0)	70 (34.3)	61 (29.9)
Missing or unknown	1028 (46.0)	5192 (46.5)	62 (30.4)	63 (30.9)

Abbreviations: HT, hormone therapy; IQR, interquartile range; DDD, defined daily doses.

**Table 2:** Association between dementia and systemic hormone therapy (HT) in the singleton and twin populations including adjustment for education and sensitivity analysis.

	Singletons			Twins		
	N (cases)	OR [95% CI]	*p*-value	N (cases)	OR [95% CI]	p-value

All dementia cases	2235			204		
No HT use		1 (ref)			1 (ref)	
Systemic HT use		1.05 [0.93–1.19]	0.422		2.10 [0.99–4.46]	0.053
Cumulative DDDs						
1–99		1.07 [0.80–1.43]	0.643			
100–499		0.88 [0.67–1.16]	0.370			
500–999		1.15 [0.85–1.55]	0.362			
1000–2000		1.08 [0.85–1.38]	0.516			
>2000		1.08 [0.89–1.31]	0.422			
Test for trend			0.696			
Age at dementia 60–79 years	744			107		
No HT use		1 (ref)			1 (ref)	
Systemic HT use		1.06 [0.97–1.16]	0.210		1.71 [0.67–4.35]	0.257
Adjusted for education	699			103		
No HT use		1 (ref)			1 (ref)	
Systemic HT use		1.07 [0.098–1.17]	0.152		1.63 [0.63–4.19]	0.310
Age at dementia ≥ 80 years	1491			97		
No HT use		1 (ref)			1 (ref)	
Systemic HT use		1.00 [0.92–1.09]	0.942		3.00 [0.81–11.1]	0.099
Adjusted for education	491			37		
No HT use		1 (ref)			1 (ref)	
Systemic HT use		1.07 [0.94–1.21]	0.300		5.82 [0.70–48.7]	0.104
Sensitivity analysis 1^a^	2235			204		
No HT use		1 (ref)			1 (ref)	
Systemic HT use		1.06 [0.94–1.21]	0.349		1.90 [0.88–4.09]	0.100
Sensitivity analysis 2^b^	2272			204		
No HT use		1 (ref)			1 (ref)	
Systemic HT use		1.04 [0.92–1.17]	0.527		2.10 [0.99–4.46]	0.053

Abbreviation: OR, odds ratio; CI, confidence interval.

a 1–2 HT prescriptions considered non-users.

b All dementia diagnoses are included regardless of age at diagnosis.

**Table 3:** Association between systemic hormone therapy (HT) and dementia diagnosis before the medical guideline alteration (1/1–2003) in both singletons and twins, including how the change in medical guidelines on prescribing systemic HT has affected the association (after 1/1–2003).

	Singletons			Twins		
	Case, N (%)	Control, N (%)	OR [95% CI]	Case, N (%)	Control, N (%)	OR [95% CI]

Dementia (1997–2011):						
Systemic HT use (1995–2002)						
Type of administration						
No HT use	1902 (82.5)	9694 (84.2)	1 (ref)	176 (80.7)	188 (86.2)	1 (ref)
Systemic HT use	403 (17.5)	1823 (15.8)	1.14 [1.01–1.28]*	42 (19.3)	30 (13.8)	2.20 [1.04–4.65]*
Systemic cumulative DDDs						
1–99			1.15 [0.85–1.56]			
100–499			1.02 [0.78–1.34]			
500–999			1.11 [0.82–1.51]			
1000–2000			1.21 [0.97–1.52]			
>2000			1.15 [0.94–1.41]			
Test for trend			p-value = 0.65			
Dementia (2005–2011): Change in the medical guidelines on systemic						
HT use						
User status						
Never-user	1016 (78.8)	5160 (80.0)	1 (ref)	78 (70.3)	88 (79.3)	1 (ref)
Previous user^a^	132 (10.2)	562 (8.7)	1.20 [0.98–1.47]	21 (18.9)	6 (5.4)	10.65 [2.08–54.54]*
Current user^b^	142 (11.0)	728 (11.3)	1.00 [0.82–1.21]	12 (10.8)	17 (15.3)	1.55 [0.43–5.56]

a Users before 1/1–2003 but not after, and dementia after 2005.

b Users after 1/1–2003.

* *p*-value <0.05.

**Table 4:** Intrapair association between systemic hormone therapy (HT) and dementia in the twin population.

All twins (incl. UZ)	*Control*		
		
*Dementia case*	*HT* +	*HT* −	

*HT* +	20	21	41
*HT* −	10	153	163
	30	174	204
OR [95% CI]	2.10 [0.95–4.99], p-value = 0.071		
**MZ TWINS**			
OR [95% CI]	6.00 [0.73–275.99], p-value = 0.125		
**DZ TWINS**			
OR [95% CI]	2.50 [0.92–7.86], p-value = 0.078		

Abbreviations: UZ, unknown zygosity; MZ, monozygotic; DZ, dizygotic.
