# Supplementary material for: Systemic hormone therapy and dementia: A nested case-control and co-twin control study
Source: Maturitas. Author manuscript; Available in PMC 2025 May 1. (PMC12044222; doi:10.1016/j.maturitas.2022.04.007)
Supplement: Supplementary Material [file NIHMS2072541-supplement-Supplementary_Material.docx]

**Supplementary Table S1**

*Danish Nationwide Health Registries*

| Danish Civil Registration System | Established in 1968 and provides each individual living permanently in Denmark with a unique personal identification number [1], which enables linkage between national registries. |
| --- | --- |
| The Danish Twin Registry | A population-based registry, which was established in the 1950s and includes information on more than 175,000 Danish twins born from 1870 to 2009 [2]. Zygosity in same-sex twin pairs has been categorized using a questionnaire with four questions on similarity, which has shown a misclassification of less than 5% [3]. |
| The Danish National Prescription Registry | Has gathered information from all retail pharmacies on all redeemed prescriptions for residents in Denmark since 1995. Data includes, among other variables, recipient ID, prescriber ID, date of dispensing, and route of administration in addition to substance, using the Anatomical Therapeutic Chemical code (ATC code) and the dispensed quantity measured in defined daily doses [4]. |
| The Danish National Patient Registry | Holds information on diagnoses from hospital admissions since 1977 and contacts with outpatient clinics and emergency departments since 1995. It has registered all patient contacts including date of admission, date of discharge, and type of diagnosis according to the WHO International Classification of Diseases (ICD) 8^th^ revision from 1977 to 1993 (ICD-8) and 10^th^ revision from 1994 (ICD-10) [5]. |

**References**

[1] C.B. Pedersen, The Danish Civil Registration System, Scandinavian journal of public health 39(7 Suppl) (2011) 22-5.

[2] D.A. Pedersen, L.A. Larsen, M. Nygaard, J. Mengel-From, M. McGue, C. Dalgard, L. Hvidberg, J. Hjelmborg, A. Skytthe, N.V. Holm, K.O. Kyvik, K. Christensen, The Danish Twin Registry: An Updated Overview, Twin research and human genetics : the official journal of the International Society for Twin Studies (2019) 1-9.

[3] L. Christiansen, H. Frederiksen, K. Schousboe, A. Skytthe, N. von Wurmb-Schwark, K. Christensen, K. Kyvik, Age- and sex-differences in the validity of questionnaire-based zygosity in twins, Twin research : the official journal of the International Society for Twin Studies 6(4) (2003) 275-8.

[4] A. Pottegard, S.A.J. Schmidt, H. Wallach-Kildemoes, H.T. Sorensen, J. Hallas, M. Schmidt, Data Resource Profile: The Danish National Prescription Registry, International journal of epidemiology 46(3) (2017) 798-798f.

[5] M. Schmidt, S.A. Schmidt, J.L. Sandegaard, V. Ehrenstein, L. Pedersen, H.T. Sorensen, The Danish National Patient Registry: a review of content, data quality, and research potential, Clinical epidemiology 7 (2015) 449-90.
